# Total Flavonoids of Rhizoma Drynariae Restore the MMP/TIMP Balance in Models of Osteoarthritis by Inhibiting the Activation of the NF-*κB* and PI3K/AKT Pathways

**DOI:** 10.1155/2021/6634837

**Published:** 2021-04-19

**Authors:** Guang-Yao Chen, Jia-Qi Chen, Xiao-Yu Liu, Yuan Xu, Jing Luo, Yi-Fei Wang, Tong-Liang Zhou, Ze-Ran Yan, Li Zhou, Qing-Wen Tao

**Affiliations:** ^1^Beijing University of Chinese Medicine, Beijing 100029, China; ^2^Department of TCM Rheumatology, China-Japan Friendship Hospital, Beijing 100029, China; ^3^Beijing Key Lab for Immune-Mediated Inflammatory Diseases, China-Japan Friendship Hospital, Beijing 100029, China

## Abstract

Total flavonoids of *Rhizoma Drynariae* (TFRD) have been shown to have beneficial effects on osteoarthritis (OA) clinically, but the mechanisms have not been elucidated. In this study, we investigated the effect of TFRD on articular cartilage in an OA rat model established by the Hulth method and in SW1353 chondrocytes induced by the proinflammatory factor interleukin-1*β* (IL-1*β*). The results showed that TFRD could alleviate the pathological changes in knee cartilage in OA model rats. In vivo, the qPCR analysis indicated that the mRNA levels of matrix metalloproteinases, MMP-1, MMP-3, and MMP-13, were decreased, while tissue inhibitor of matrix metalloproteinases- (TIMP-) 4 was increased in cartilage, and these changes could be partially prevented by TFRD. In vitro experiments showed that IL-1*β* could significantly increase the expression of MMP-1, MMP-3, and MMP-13 and decrease the expression of TIMP-4 in SW1353 cells at the mRNA and protein levels. TFRD could increase the expression of MMP-3 and MMP-13 and decrease the expression of TIMP-4. Transfection of siRNA and addition of pathway inhibitors were used to clarify that inhibition of NF-*κB* and PI3K/AKT pathway decreased MMP-1, MMP-3, and MMP-13 and increased TIMP-4 expression. We also found that in IL-1*β*-induced SW1353 cells, TFRD pretreatment had a modest inhibitory effect on p-AKT (Ser473) and reversed the increase of nuclear factor kappa-B (NF-*κB*) p65 in nuclear fraction and the decrease of inhibitor of NF-*κB*(*IκB*)-*α* in the cytosolic fraction. Further immunofluorescence confirmed that TFRD can inhibit IL-1*β*-induced NF-*κB* p65 translocation to the nucleus to some extent. In conclusion, TFRD showed chondroprotective effects by restoring the MMP/TIMP balance in OA models by suppressing the activation of the NF-*κB* and PI3K/AKT pathways.

## 1. Introduction

Osteoarthritis (OA) is a degenerative joint disease characterized by progressive cartilage degradation and is one of the most leading causes of disability in elderly individuals [1]. Evidence shows that OA is closely related to age, obesity, and joint trauma [2]. With increases in the aging and obese populations, OA has become a major public health issue worldwide [3].

The main pathological feature of OA is cartilage destruction, and disequilibrium of cartilage synthesis and degradation appears earlier in the course of the disease than structural changes [4]. Matrix metalloproteinases (MMPs) are a class of metal ion-dependent endopeptidases that are closely related to cartilage degradation through their ability to break down the extracellular matrix (ECM) [5]. Tissue inhibitors of matrix metalloproteinases (TIMPs) can specifically inhibit MMPs activity and maintain homeostasis in healthy cartilage [6]. After being stimulated by proinflammatory factors, chondrocytes synthesize and secrete massive quantities of MMPs while inhibiting the expression of TIMPs [7]. The proinflammatory cytokine interleukin-1*β* (IL-1*β*) can be produced by a series of cells, such as chondrocytes and synovial cells [8]. IL-1*β* can also significantly upregulate MMPs expression in chondrocytes, which has a critical effect on the progression of OA, especially in the early stage [9].

OA causes severe damage; however, effective and safe drugs to treat OA are still lacking [10]. Nonsteroidal anti-inflammatory drugs (NSAIDs) are used to lessen the related symptoms of OA. Long-term use of NSAIDs can cause certain side-effects, and alternative drugs still require further study [11, 12]. Currently, plant-derived agents have drawn considerable attention as reliable and efficient alternative therapies for the treatment of OA [13].

Rhizoma Drynariae is the root of *Drymotaenium fortunei* (Kze.) J. Smith and has been used in traditional Chinese medicine for a long time to treat bone-related diseases, such as fractures, joint pain, and osteoporosis [14]. In China, total flavonoids of Rhizoma Drynariae (TFRD) has been approved by China National Medical Products Administration (NMPA) for treatment of osteoporosis. Osteoporosis and osteoarthritis were common comorbidities among the elderly and it was found that some of the symptoms of osteoarthritis, such as pain of the knee joint and limb motor dysfunction, improved significantly after treatment with TFRD for osteoporosis. Furthermore, some clinical studies have shown the beneficial effect of TFRD on OA [15]. However, the mechanisms by which TFRD affects OA have not been elucidated. To explore the impact of TFRD in articular cartilage and the specific mechanisms, we established a rat knee OA model using the Hulth method, and IL-1*β*-induced SW1353 chondrocytes were used for in vitro OA studies.

## 2. Materials and Methods

### 2.1. Reagents and Antibodies

The following reagents and antibodies were used: SYBR Green Realtime PCR Master Mix (QPK-201, Toyobo, Japan), 0.25% Trypsin-EDTA (Gibco, USA), penicillin-streptomycin (Gibco, USA), fetal bovine serum (ScienCell, USA), Leibovitz's L-15 medium (Gibco, USA), polyvinylidene difluoride (PVDF) membranes (Millipore, USA), electrochemiluminescence reagents (Millipore, USA), interleukin-1*β* (Peprotech, USA), dexamethasone (Sigma, USA), phosphate-buffered saline (PBS, HyClone, USA), 10% SDS-polyacrylamide precast gels (Applygen Technologies Inc., China), Lipofectamine 2000 (Invitrogen, USA), Opti-MEM (Gibco, USA), primary and secondary antibody diluent (Applygen Technologies Inc., China), 5× loading buffer (Applygen Technologies Inc., China), MTS (Promega, USA), RIPA lysis buffer (Solarbio Life Sciences, China), membrane blocking solution (Solarbio Life Sciences, China), phenylmethanesulfonyl fluoride (PMSF) (Solarbio Life Sciences, China), DAPI solution (Solarbio Life Sciences, China), MK-2206 2HCl (Selleck Chemicals, USA), JSH-23 (Selleck Chemicals, USA), MMP-3 and TIMP-4 enzyme-linked immunosorbent assay (ELISA) kits (R&D, USA), MMP-1 and MMP-13 ELISA kits (Novus Biologicals, USA), polymerase chain reaction (PCR) primers for MMP-1, MMP-3, MMP-13, TIMP-4, NF-*κB* p65, and *β*-actin (Tingke, China; primer sequences are listed in Table 1), AKT siRNA, NF-*κB* p65 siRNA (Tingke, China; sequences are listed in Table 2), MMP-1 antibody (10371-2-AP, Proteintech, USA), MMP-3 antibody (ab53015, Abcam, UK), MMP-13 antibody (ab39012, Abcam, UK), TIMP-4 antibody (BS1477, Bioworld Technology, USA), NF-*κB* p65 antibody (8242T, Cell Signaling, USA), *IκB*-*α* antibody (4814T, Cell Signaling, USA), AKT antibody (ab8805, Abcam, UK), p-AKT (S473) antibody (4060S, Cell Signaling, USA), *β*-Actin antibody (TA-09, Zhongshan Jinqiao Biotechnology, China), Lamin B1 antibody (YT5108, Immunoway, USA), horseradish peroxidase- (HRP-) conjugated goat anti-mouse IgG (ZB-5305, Zhongshan Jinqiao Biotechnology, China), HRP-conjugated goat anti-rabbit IgG (ZB-2301, Zhongshan Jinqiao Biotechnology, China), and Alexa Fluor 488-conjugated Goat anti-Rabbit IgG (H + L) (ZF-0511, Zhongshan Jinqiao Biotechnology, China).

### 2.2. Preparation of TFRD

**Figure 1:**
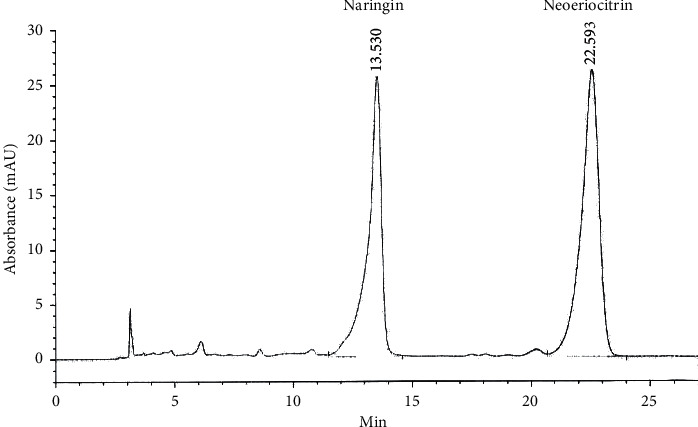
Chromatographic fingerprint of TFRD.

TFRD was purchased from Beijing Qihuang Pharmaceutical Co., Ltd., China. TFRD extraction was based on the following protocol [16]: the dried root of *Drymotaenium fortunei* (Kze.) J. Smith was cut into small pieces; tenfold distilled water was added; then, they were boiled at 100°C and decocted for an hour, then filtered, and centrifuged. The process was repeated twice, and the liquid obtained after three repeated extractions was mixed. The filtered solution was passed through a macroporous adsorption resin column to adsorb the TFRD. The column was washed with water, and elution was performed with 70% ethanol. Eluent solutions were concentrated and vacuum-dried to obtain the TFRD. Chromatographic analysis showed that the main components of TFRD were naringin (42.45%) and neoeriocitrin (57.55%), which had retention times of 13.53 min and 22.59 min, respectively (Figure 1).

The daily dose of TFRD approved by the NMPA for the treatment of osteoporosis is 0.6 g, which was used as a reference for the intervention of OA treatment. The dosage of TFRD was calculated as 10 mg/kg according to the adult weight of 60 kg. In the animal experiments, the body surface area method was used for dosimetry calculations, and the amount used in rats is 63 mg/kg. For the convenience of calculation, the rats were given 1 mL solution per 100 g body weight by gavage, so the drug concentration was 6.3 mg/ml. For the in vitro experiments, TFRD was mixed with sterile PBS; syringe filters (0.22 *μ*m) were used to filter the drug solutions to ensure sterility before use.

### 2.3. Animals and Treatments

Six-week-old male SD rats were purchased from SPF (Beijing) Biotechnology Co., Ltd. All rats were maintained in accordance with the National Standards for Laboratory Animals of China (GB 14925-2010).

A total of 36 rats were divided randomly and equally into three groups (*n* = 12/group): the normal blank control group, OA model group, and TFRD-treated group. Knee OA was induced in the OA model group and TFRD-treated group by the Hulth method under pentobarbital anesthesia [17]. The rats received an intraperitoneal injection of penicillin (200,000 U/kg) for three days after surgery to prevent bacterial infection. TFRD was dissolved in saline and was then administered by gavage to the TFRD-treated group. The blank control group and OA model group received the same volume of saline.

Oral gavage was performed every day and lasted for eight weeks, according to our previous study. After the day of the last gavage treatment, all rats were euthanized by excessive pentobarbital sodium administration, and the left knee joint was collected (6 from each group were randomly selected for pathological assessment, and the other 6 were used for PCR analysis).

### 2.4. Articular Cartilage Pathological Assessment

Sections of the knee joint were stained with hematoxylin-eosin (HE) to visualize cartilage destruction. The joint was fixed with 4% formaldehyde for 72 h and decalcified in a 10% EDTA solution for 8 weeks before being embedded in wax blocks. Tissues were cut into slices (7 *µ*m) and stained with HE. The evaluation was performed by light microscopy according to Mankin's grading system [18].

### 2.5. Cell Culture

SW1353 cells (derived from human chondrosarcoma) were obtained from the American Type Culture Collection. The cells were cultured at 37°C in a humidified incubator in complete Leibovitz's L-15 medium (containing 10% fetal bovine serum to provide extra nutrition and penicillin and streptomycin to prevent bacterial contamination). For the MTS assay, cells were seeded in a 96-well plate. For PCR and Western blot analyses, cells were seeded in 6-well plates. Leibovitz's L-15 medium was used to support cell growth in an environment without CO_2_; thus, the culture bottle and plate were sealed as tightly as possible to minimize gas exchange with outside air.

### 2.6. MTS Assay

The viability of cultured cells was measured by the MTS assay. SW1353 cells were diluted to a concentration of 2 × 10^5^ cells/mL and seeded at 100 *µ*l per well in a 96-well plate (2 × 10^4^ cells per well). TFRD solution was added to each well to final concentrations of 300, 400, 500, 600, 700, 800, and 900 mg/L in the presence or absence of 10 ng/mL IL-1*β*. After 12 h of culture, the medium was discarded, and the cells were washed twice with PBS. Then, 100 *µ*l of fresh complete Leibovitz's L-15 medium was added to each well. The cells were further cultured for 2 h, and then 20 *µ*l of MTS was added. Incubation was continued for an additional 2 h. The optical density (OD) of each well was measured by a microplate reader (Molecular Devices, USA) at 490 nm. The following formula was used to calculate cell viability: viability (%) = 100 × (OD of treated sample-OD of medium)/(OD of control sample-OD of medium).

### 2.7. siRNA Transfections

SW1353 cells were seeded in 6-well plates at a density of 4×105 cells/well. RNase-free water and Opti-MEM were used to dilute siRNA to appropriate concentrations. Subsequently, cells were transfected with siRNA using Lipofectamine 2000, according to the manufacturer's protocol. After 24 h, cell lysates were collected for PCR analysis to determine the expression of target genes.

### 2.8. RNA Isolation and PCR

A column cartilage RNA extraction kit (Tiandz, China) was used to isolate total RNA from articular cartilage, while total RNA was extracted from cultured cells using a HiPure Total RNA mini kit (Magen, China). Samples were lysed in RL buffer, and the mixture was added to a gDNA filter mini column to filter out the DNA. The column was discarded, and 70% ethanol solution was added to the filtrate (equivolume mixes). Then, the mixture was added to a HiPure RNA mini column and centrifuged, and the RNA was adsorbed on the column. The column was washed three times with RW buffer, and 40 *µ*l of RNase-free water was added to the column, which was then centrifuged. Then, the total RNA solution was obtained and stored at −80°C. The concentration and quality of the RNA were quantified using a NanoDrop spectrophotometer (Thermo Scientific, USA). cDNA was obtained by a reverse transcription system (A3500, Promega, USA) according to the manufacturer's instructions and was then stored at −20°C until use.

qPCR was performed with SYBR Green Realtime PCR Master Mix according to the manufacturer's instructions. The relative expression was calculated based on the ΔΔ*Ct* method using the following equations: ΔΔ*Ct* = (Ct target–Ct actin) treatment–(Ct target–Ct actin) control and fold change = 2^−ΔΔ*Ct*^.

### 2.9. Western Blotting

Total protein extraction was performed using RIPA buffer. After being washed with PBS, the cells were lysed with 250 *µ*l of RIPA buffer (containing 1% PMSF and 1% phosphatase inhibitor) per well, agitated gently, and placed on ice for 25 min. Then, the lysed cells were centrifuged at 10000 r/min for 5 min, and the supernatant was separated. The total protein concentration was measured by a BCA kit, and all samples were diluted to equal concentrations and then stored at −80°C.

Protein solution (40 *µ*l) was combined with 5× loading buffer (10 *µ*l) and boiled at 100°C for 5 min. The mixture was resolved by 10% SDS-PAGE (120 V for the concentrated gel and 80 V for the separation gel). Subsequently, the Sandwich method was used to transfer the proteins onto a PVDF membrane (70 V for 60 min). The membrane was incubated in a dedicated Western blot blocking solution for 1 h, and then, the primary antibody was added and incubated for 12 h at 4°C. Corresponding HRP-labeled secondary antibodies were added and incubated at room temperature for 1.5 h, and the membranes were extensively washed with TBST in between the additions. ECL luminescent solution (a 1 : 1 mixture of liquid A and liquid B) was added, and the membrane was imaged with a chemiluminescence system (BioRad, USA).

### 2.10. ELISA

The cell culture supernatants were collected and centrifuged, and samples were diluted with PBS to a suitable concentration before analysis. The concentrations of MMP-1, MMP-3, MMP-13, and TIMP-4 were measured by ELISA kits according to the manufacturer's instructions. Curve Expert 1.3 software was used to generate the standard curves for the ELISA results.

### 2.11. Immunofluorescence

Cells were washed with PBS before being fixed with 4% paraformaldehyde for 15 min. Then, 0.1% Tritax-100 was used to permeabilize the cell membranes. NF-*κB* p65 antibody (1 : 400 dilution) was added to the plate and incubated overnight at 4°C. After discarding the NF-*κB* p65 antibody dilutions, Alexa Fluor 488-Conjugated goat anti-Rabbit IgG (H + L) (1 : 200 dilution) and DAPI solution were added. Immunofluorescence was observed by fluorescence microscope (Zeiss, Germany).

### 2.12. Statistical Analysis

Continuous variables are presented as the mean values ± standard deviation (SD). Student's *t*-test was used to assess the differences between the two groups. ANOVA and Dunnett's test were used for comparisons of multiple groups (GraphPad Prism 4). A *p*-value <0.05 was considered to indicate a statistically significant difference.

## 3. Results

### 3.1. TFRD Alleviates OA-Associated Cartilage Damage

**Figure 2:**
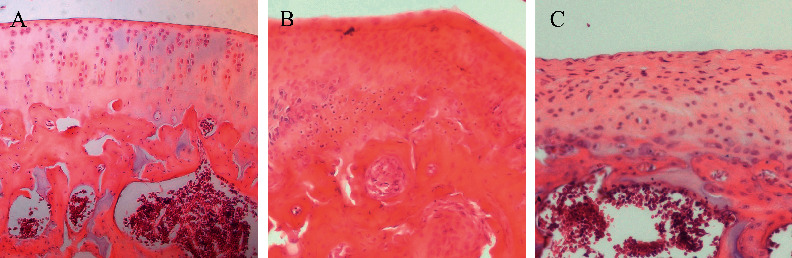
Pathological changes in the different groups (hematoxylin-eosin staining). (a) Blank control group; (b) OA model group (established with the Hulth method); (c) TFRD-treated group (treated with total flavonoids of *Rhizoma Drynariae* (TFRD) after model induction with the Hulth method).

**Figure 3:**
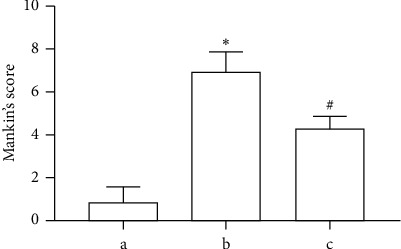
Mankin's scoring was based on the hematoxylin-eosin (HE) staining results. (a) Blank control group; (b) OA model group (established with the Hulth method); (c) TFRD-treated group (treated with total flavonoids of *Rhizoma Drynariae* (TFRD) after model induction with the Hulth method. The data are expressed as the mean ± standard deviation (^*∗*^*p* < 0.01 compared with Group a; ^#^*p* < 0.01 compared with Group b).

To explore the effect of TFRD on OA-associated cartilage destruction, we applied the Hulth method to establish a knee OA model in rats and used TFRD as a pharmacological intervention. The results suggested that TFRD could significantly alleviate articular cartilage damage in knee OA (Figure 2) and significantly reduce the Mankin's score (*p* < 0.01, Figure 3).

### 3.2. TFRD Inhibits the mRNA Expression of MMP-1, MMP-3, and MMP-13 and Enhances TIMP-4 Expression in OA Rat Cartilage

**Figure 4:**
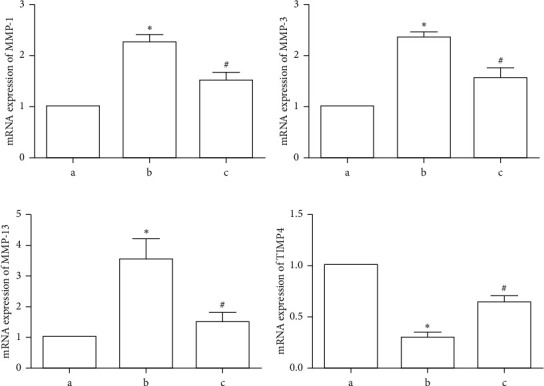
Effects of total flavonoids of *Rhizoma Drynariae* (TFRD) on MMP-1, MMP-3, MMP-13, and TIMP-4 mRNA expression in articular cartilage in the different groups. Group a, blank control group; Group b, OA model group (established with the Hulth method); Group c, TFRD-treated group (treated with TFRD after model induction with the Hulth method). The data are derived from six independent experiments and expressed as the mean ± standard deviation (^*∗*^*p* < 0.05 compared with Group a; ^#^*p* < 0.05 compared with Group b).

Total RNA was extracted from the articular cartilage of rats in the three groups. Compared with those of the normal blank control group, the mRNA levels of MMP-1, MMP-3, and MMP-13 were increased, while TIMP-4 was reduced in the OA model group at 8 weeks after model establishment (*p* < 0.05). TFRD intervention prevented these changes to some extent (*p* < 0.05, Figure 4).

### 3.3. Effects of TFRD on SW1353 Cell Viability

**Figure 5:**
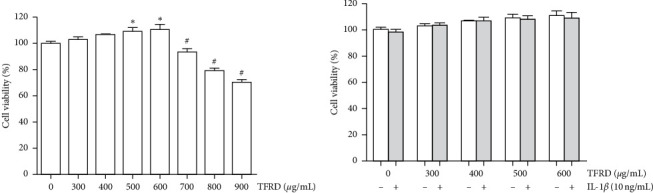
The viability of SW1353 cells after treatment with different concentrations of total flavonoids of *Rhizoma Drynariae* (TFRD) and 10 ng/mL IL-1*β*. (a) SW1353 cells were treated with different concentrations of TFRD alone for 12 h. (b) SW1353 cells were treated with different concentrations of TFRD with or without 10 ng/mL IL-1*β* for 12 h. The data are derived from six independent experiments and expressed as the mean ± standard deviation (^*∗*^*p* < 0.05: cell viability increased compared with the control group; ^#^*p* < 0.05: cell viability decreased compared with the control group).

To determine the appropriate treatment concentration for SW1353 cells, we treated the cells with 300, 400, 500, 600, 700, 800, and 900 *μ*g/mL TFRD for 12 h. The MTS assay results indicated that compared with the control group, treatment with TFRD at 500 and 600 *μ*g/mL promoted cell viability(*p* < 0.05), but significant cytotoxicity was observed at concentrations of 700, 800, and 900 *μ*g/mL (*p* < 0.05, Figure 5(a)). Subsequently, 10 ng/mL IL-1*β* was added to the groups in which the TFRD concentration was lower than 600 *μ*g/mL. The results showed that 10 ng/mL IL-1*β* induced no side-effects (*p* > 0.05, Figure 5(b)). Therefore, the maximum treatment concentration of TFRD in SW1353 cells was 600 *μ*g/mL in subsequent experiments, with or without 10 ng/mL IL-1*β*.

### 3.4. TFRD Inhibits the mRNA Expression of MMP-3 and MMP-13 and Enhances TIMP-4 Expression in IL-1*β*-Induced SW1353 Cells

**Figure 6:**
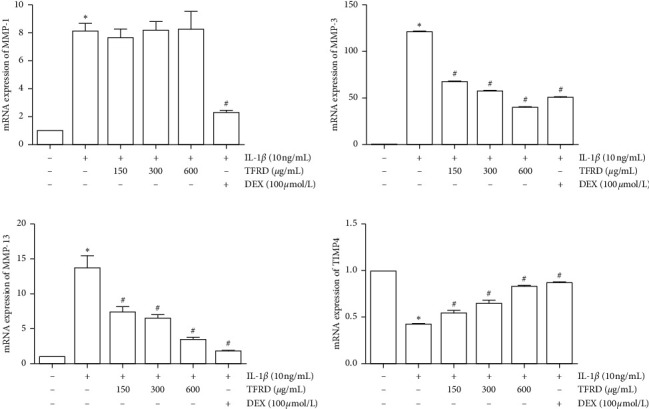
Inhibition of MMP-3 and MMP-13 and promotion of TIMP-4 mRNA expression in IL-1*β*-induced SW1353 cells by total flavonoids of *Rhizoma Drynariae* (TFRD). Cells were pretreated with 150, 300, or 600 *μ*g/mL TFRD or 100 *μ*mol/L dexamethasone (DEX) for 1 h and then stimulated with 10 ng/mL IL-1*β* for 12 h. The data are derived from three independent experiments and expressed as the mean ± standard deviation. (^*∗*^*p* < 0.05 compared with the control group; ^#^*p* < 0.05 compared with the IL-1*β*-treated group).

qPCR was performed to investigate whether TFRD regulates the IL-1*β*-induced expression of the OA-related genes, MMP-1, MMP-3, MMP-13, and TIMP-4, in SW1353 cells. The results showed that the mRNA expression of MMP-1, MMP-3, and MMP-13 was dramatically increased, while the mRNA expression of TIMP-4 was decreased in IL-1*β*-induced SW1353 cells (*p* < 0.05, Figure 6). These results showed an apparent dose-dependent response to different concentrations of TFRD (*p* < 0.05, Figures 5(b)–5(d)). However, the different concentrations of TFRD exhibited no significant effect on MMP-1 mRNA expression (*p* > 0.05), whereas the positive control drug dexamethasone (100 *μ*mol/L) obviously inhibited the mRNA expression of MMP-1 (*p* < 0.05). These results suggest that TFRD modulated the expression of MMPs and TIMP-4 at the transcriptional level to a certain extent.

### 3.5. TFRD Inhibits the Protein Expression of MMP-3 and MMP-13 and Enhances TIMP-4 Expression in IL-1*β*-Induced SW1353 Cells

**Figure 7:**
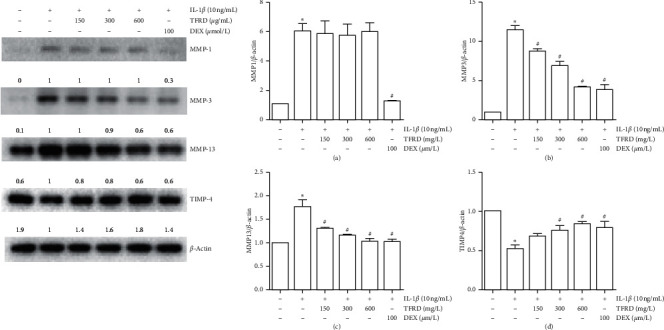
Inhibition of MMP-3 and MMP-13 and promotion of TIMP-4 protein expression in IL-1*β*-induced SW1353 cells by total flavonoids of *Rhizoma Drynariae* (TFRD). Before stimulation with 10 ng/mL IL-1*β* for 12 h, cells were pretreated with 150, 300, or 600 *μ*g/mL TFRD or 100 *μ*mol/L dexamethasone (DEX) for 1 h. The cells were lysed, and MMP-1, MMP-3, MMP-13, and TIMP-4 levels were measured by Western blotting. The data are derived from three independent experiments and expressed as the mean ± standard deviation. (^*∗*^*p* < 0.05 compared with the control group; ^#^*p* < 0.05 compared with the IL-1*β*-treated group).

Western blotting was used to measure the protein expression of MMP-1, MMP-3, MMP-13, and TIMP-4 in IL-1*β*-induced SW1353 cells to evaluate the effects of TFRD. The results showed that compared to the protein levels in the control group, the protein levels of MMP-1, MMP-3, and MMP-13 in IL-1*β*-induced SW1353 cells were obviously increased, and the level of TIMP-4 was decreased (*p* < 0.05). When IL-1*β*-induced SW1353 cells were treated with TFRD, the protein expression of MMP-3 and MMP-13 was reduced and the protein expression of TIMP-4 was enhanced (*p* < 0.05). We also observed that these changes showed a clear dose-dependent effect (Figure 7).

### 3.6. TFRD Inhibits the Secretion of MMP-3 and MMP-13 from IL-1*β*-Induced SW1353 Cells

**Figure 8:**
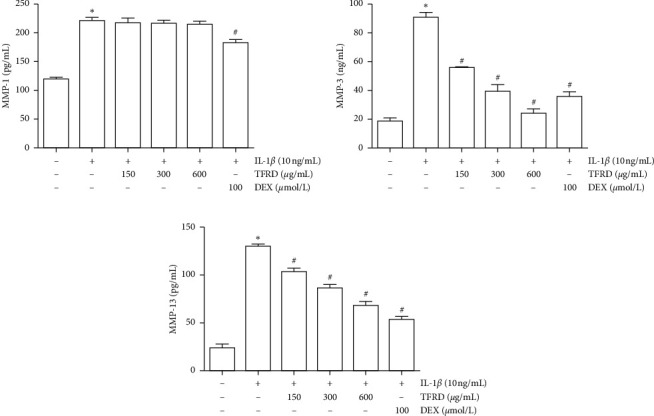
Inhibition of MMP-3 and MMP-13 protein expression in IL-1*β*-induced SW1353 cells by total flavonoids of *Rhizoma Drynariae* (TFRD). Before stimulation with 10 ng/mL IL-1*β* for 12 h, cells were pretreated with 150, 300, or 600 *μ*g/mL TFRD or 100 *μ*mol/L dexamethasone (DEX) for 1 h. Subsequently, the supernatants of the cultured cells were collected, and MMP-1, MMP-3, and MMP-13 levels were measured by ELISA. The data are derived from three independent experiments and expressed as the mean ± standard deviation (^*∗*^*p* < 0.01 compared with the control group; ^#^*p* < 0.05 compared with the IL-1*β*-treated group).

MMP-1, MMP-3, MMP-13, and TIMP-4 release was measured by ELISA in the supernatant of cells cultured for 12 h. The results showed that IL-1*β* promoted the release of MMP-1, MMP-3, and MMP-13 from SW1353 cells (*p* < 0.01). Pretreatment with different concentrations of TFRD suppressed the levels of MMP-3 and MMP-13 in the culture supernatant, which were similar to the Western blot results (Figure 8). Additionally, we attempted to measure TIMP-4 protein expression in cell supernatant by ELISA but were unsuccessful.

### 3.7. Effects of PI3K/AKT and NF-*κB* in the MMP/TIMP Balance

**Figure 9:**
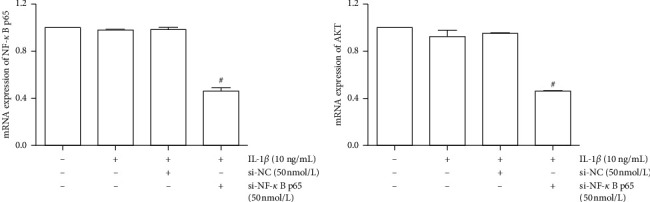
Inhibition of NF-*κB* p65 and AKT mRNA expression in IL-1*β*-induced SW1353 cells by si-NF-*κB* and si-AKT. The data are derived from three independent experiments and expressed as the mean ± standard deviation (^#^*p* < 0.01 compared with the IL-1*β*-treated group).

**Figure 10:**
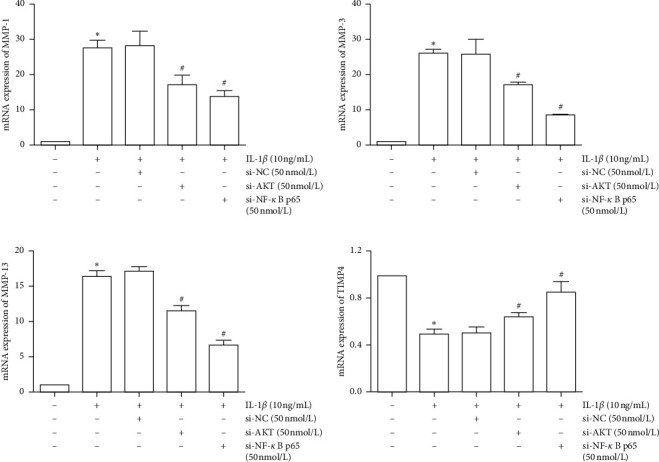
Inhibition of MMP-1, MMP-3, MMP-13, and TIMP4 mRNA expression in IL-1*β*-induced SW1353 cells by si-AKT and si-NF-*κB*. The data are derived from three independent experiments and expressed as the mean ± standard deviation (^*∗*^*p* < 0.01 compared with the control group; ^#^*p* < 0.05 compared with the IL-1*β*-treated group).

**Figure 11:**
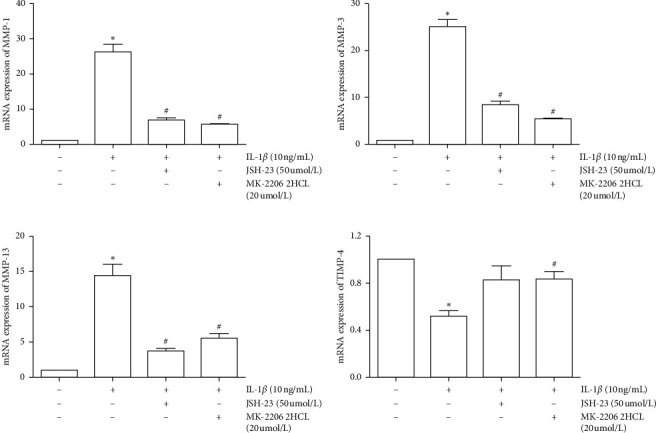
Inhibition of MMP-1, MMP-3, MMP-13, and TIMP4 mRNA expression in IL-1*β*-induced SW1353 cells by JSH-23 and MK-2206. The data are derived from three independent experiments and expressed as the mean ± standard deviation (^*∗*^*p* < 0.05 compared with the control group; ^#^*p* < 0.05 compared with the IL-1*β*-treated group).

To ensure the role of PI3K/AKT and NF-*κB* pathway in the MMP/TIMP balance, we used siRNA and pathway inhibitors for confirmation. First, we, respectively, used si-NF-*κB* p65 or si-AKT to inhibit the expression of NF-*κB* p65 and AKT mRNA (Figure 9). The PI3K/AKT and NF-*κB* pathways were inhibited by inhibiting the expression of AKT and NF-*κB* p65 mRNA. Results showed that inhibition of the PI3K/AKT and NF-*κB* pathway can reduce the expression of MMP-1, MMP-3, and MMP-13, as well as promote the expression of TIMP4 (Figure 10). Furthermore, we used MK-2206 (a phosphorylation inhibitor of AKT) to inhibit the activity of AKT and JSH-23 (a transcriptional activity inhibitor of NF-*κB*) to inhibit the activity of the NF-*κB* pathway, which can also reduce the expression of MMP-1, MMP-3, and MMP-13 and promote the expression of TIMP-4 (Figure 11).

### 3.8. Effects of TFRD on IL-1*β*-Induced Activation of the PI3K/AKT Pathway

**Figure 12:**
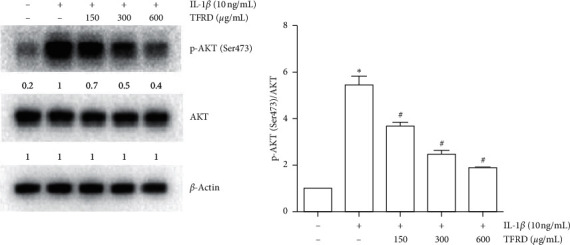
Inhibition of p-AKT levels in IL-1*β*-induced SW1353 cells by total flavonoids of *Rhizoma Drynariae* (TFRD). Before stimulation with 10 ng/mL IL-1*β* for 12 h, cells were pretreated with 150, 300, or 600 *μ*g/mL TFRD for 1 h. Subsequently, the cells were lysed, and AKT and p-AKT (Ser473) were measured by Western blotting. The data are derived from three independent experiments and expressed as the mean ± standard deviation. (^*∗*^*p* < 0.01 compared with the control group; ^#^*p* < 0.05 compared with the IL-1*β*-treated group).

Previous research has shown that the PI3K/AKT signaling pathway can be affected by inflammation and plays an essential role during the development of OA. The present study revealed that IL-1*β* did not affect the protein expression of AKT but clearly promoted the phosphorylation of AKT (Ser473) (*p* < 0.01). Pretreatment with TFRD had a modest inhibitory effect on p-AKT (Ser473) levels (*p* < 0.05), and a dose-dependent relationship was observed (Figure 12).

### 3.9. Effects of TFRD on IL-1*β*-Induced Activation of the NF-*κB* Pathway

**Figure 13:**
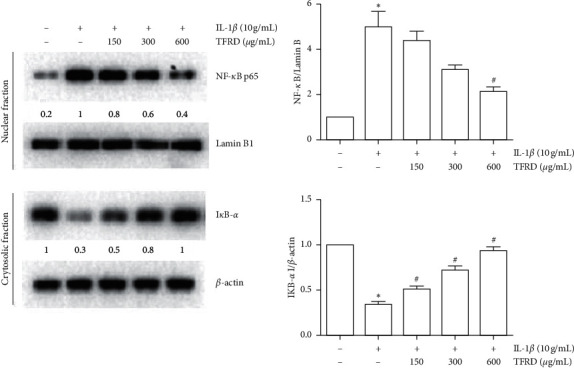
Effects of total flavonoids of *Rhizoma Drynariae* (TFRD) on NF-*κB* p65 and *IκB*-*α* levels in IL-1*β*-induced SW1353 cells. Before stimulation with 10 ng/mL IL-1*β* for 30 min, cells were pretreated with 150, 300, or 600 *μ*g/mL TFRD for 1 h. Total cytosolic and nuclear proteins were extracted. *IκB*-*α* in the cytosol and NF-*κB* p65 in the nucleus were measured by Western blotting. Lamin B and *β*-actin were used as internal references for the nuclear and cytoplasmic fractions, respectively. The data are derived from three independent experiments and expressed as the mean ± standard deviation. (^*∗*^*p* < 0.05 compared to the control group; ^#^*p* < 0.05 compared to the IL-1*β*-treated group).

**Figure 14:**
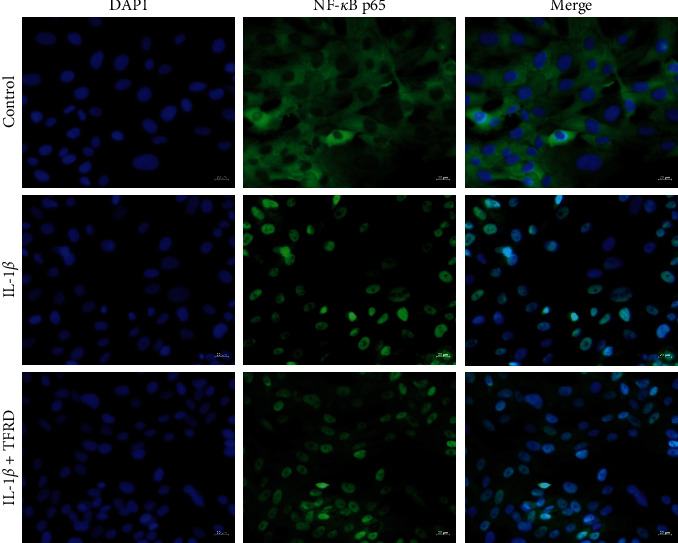
The cells were pretreated with TFRD (600 *µ*g/ml) for 1 h before IL-1*β* treatment (10 ng/ml). After 30 min of incubation, the localization of NF-*κB* p65 was visualized with fluorescence microscopy after immunofluorescence stained with anti-NF-*κB* p65 antibody (green). The cells were also stained with DAPI to visualize the nuclei (blue).

Proinflammatory cytokine activation of the NF-*κB* pathway is generally accompanied by a transient decrease in *IκB*-*α* in the cytosol and an increase in NF-*κB* p65 in the nucleus. After SW1353 cells were stimulated with IL-1*β* for 30 min, total cytosolic and nuclear proteins were extracted using a specific kit (Solarbio Life Sciences, China). The results showed that in IL-1*β*-stimulated SW1353 cells, NF-*κB* p65 was increased in the nucleus and *IκB*-*α* was decreased in the cytosol compared with those of untreated cells (*p* < 0.05), indicating that the NF-*κB* pathway was activated. We found that pretreatment with TFRD prevented these changes to some extent (*p* < 0.05, Figure 13). In further study, immunofluorescence was used to examine the shuttle of NF-*κB* p65 between cytoplasm and nucleus. The results showed that NF-*κB* p65 was significantly transferred to the nucleus after the stimulation of IL-1*β*, and TFRD pretreatment can prevent this change to some extent (Figure 14).

## 4. Discussion

In this study, the chondroprotective effect of TFRD on articular cartilage in a rat model of OA was investigated. We also investigated whether TFRD could restore the balance of MMPs/TIMPs in SW1353 cells induced by proinflammatory factors IL-1*β* in vitro and verified these effects on related pathways.

ECM degradation is a critical process that leads to the occurrence and development of OA [19]. MMPs, especially MMP-1, MMP-3, and MMP-13, exert enormous effects on the degradation of cartilage ECM. MMP-1 and MMP-13 are closely related to cartilage collagen dissolution, which is a key protein hydrolysis event in joint diseases that is essentially irreversible [20]. MMP-3 can activate other MMPs through the proteolysis of pro-MMPs in the extracellular space [21]. TIMPs, including TIMP-1, TIMP-2, TIMP-3, and TIMP-4, are inhibitors of MMPs, and the balance between MMPs and TIMPs is critical in normal cartilage [22]. The reduction of TIMPs can increase the activity of MMPs and accelerate the degradation of ECM [23]. MMPs/TIMP balance can be disrupted by the inflammatory factor in cartilage tissue and ultimately lead to OA [24]. TIMP-1 and TIMP-4 were found to have decreased expression in OA chondrocytes and be involved in the MMPs/TIMP balance of cartilage [25]. Animal experiments have shown that TFRD can alleviate the pathological changes in knee cartilage in OA model rats. Further experiments indicated that in cartilage, MMP-1, MMP-3, and MMP-13 mRNA levels were decreased, whereas TIMP-4 was increased. In vitro experiments showed that IL-1*β* could significantly increase both the mRNA and protein levels of MMP-1, MMP-3, and MMP-13 in SW1353 cells, which was similar to the results of previous studies [26, 27]. IL-1*β* binds to receptors on the cell membrane and activates relevant pathways, acting to proinflammatory response rapidly. However, most drugs could exert effects only after being absorbed into cells, so in this experiment, SW1353 cells were pretreated with TFRD for 1 h. The results suggest that TFRD pretreatment dose-dependently inhibited MMP-3 and MMP-13 levels while increasing TIMP-4 levels in IL-1*β*-induced SW1353 cells.

NF-*κB* is a crucial transcription factor that controls many inflammatory changes [28]. Studies have shown that the development of OA is accompanied by NF-*κB* pathway activation [29, 30]. In the quiescent state, NF-*κB* exists in a nonactivated state in the cytoplasm after binding to *IκB* [31]. When cells are stimulated by various factors, such as IL-1*β* and TNF-*α*, the resulting signaling cascade rapidly activates the phosphorylation of *IκB* kinase in the cytoplasm, which ultimately results in the ubiquitination and subsequent degradation of *IκB* [32]. Degradation of the inhibitory factor *IκB* leads to the activation of NF-*κB*, which translocates to the nucleus to promote target gene expression. This process ultimately leads to the activation of MMPs, which are considered potential targets for OA therapy [29]. As a classic signaling pathway, PI3K/AKT is involved in numerous pathological processes, such as metastasis and proliferation of tumor cells and the development of insulin resistance [33, 34]. Additionally, the PI3K/AKT signaling pathway is important in inflammation by promoting the expression of many inflammatory products [35]. After stimulation by inflammatory factors, PI3K is activated through a conformational change, which leads to AKT phosphorylation and promotes the overexpression of MMPs [36].

Stimulation of SW1353 cells with IL-1*β* leads to the nuclear translocation of NF-*κB* p65, rapid degradation of *IκB*-*α* in the cytoplasm, and the phosphorylation of AKT (Ser473). These key events indicate activation of the NF-*κB* and PI3K/AKT signaling pathways. Pretreatment with TFRD normalized the *IκB*-*α* levels in the cytoplasm while suppressing IL-1*β*-dependent nuclear translocation of NF-*κB* p65 and phosphorylation of AKT. These results suggest that the NF-*κB* and PI3K/AKT signaling pathways are involved in the IL-1*β*-induced inflammatory response and inhibited by TFRD to a certain extent.

In this study, TFRD significantly alleviated articular cartilage damage in the knee in the context of OA. We demonstrated that TFRD effectively inhibited the expression of MMPs while enhancing the expression of TIMP-4 in IL-1*β*-induced SW1353 cells and articular chondrocytes in an OA rat model, and these factors play crucial roles during OA progression. In addition, these effects are mediated in part by regulating the NF-*κB* and PI3K/AKT pathways. These results indicate the potential of TFRD as an alternative drug for treating and preventing OA.

There are also some limitations in this study. The model established by the Hulth method is a surgical disruption of the joint structure, which is a model of secondary OA. This model does not reflect all the clinical characteristics of OA, and it may be necessary to further use primary OA model to explore the treatment mechanism of TFRD on OA. At the same time, MMPs and TIMPs are involved in the regulation of cartilage matrix degradation, and collagen II (COL-II), a disintegrin and metalloproteinase with thrombospondin motifs (ADAMTS), and tissue plasminogen activator (TPA) are also important components of cartilage matrix metabolism. However, they remain to be established in future experiments for TFRD inhibition of cartilage degradation.

## Figures and Tables

**Table 1:** Prime sequences for quantitative real-time PCR.

Gene name	Sequence (5′-to-3′)
Human MMP-1 sense	CTGTTTTCTGGCCACAACTG
Human MMP-1 antisense	GGAAGCCAAAGGAGCTGTAG
Human MMP-3 sense	TTGGCCATCTCTTCCTTCAG
Human MMP-3 antisense	GAAACCTAGGGTGTGGATGC
Human MMP-13 sense	GTGCCCTTCTTCACACAGAC
Human MMP-13 antisense	AGAGCAGACTTTGAGTCATTGC
Human TIMP-4 sense	CACTCGGCACTTGTGATTCG
Human TIMP-4 antisense	GTTTGATTTCATACCGGAGCA
Human NF-*κB* p65 sense	AGGAGCACAGATACCACCAAGACC
Human NF-*κB* p65 antisense	AAGCAGAGCCGCACAGCATTC
Human AKT sense	CACGTCGGAGACTGACACC
Human AKT antisense	GCTGGCCGAGTAGGAGAAC
Human *β*-actin sense	TGGTGAAGACGCCAGTGGA
Human *β*-actin antisense	GCACCGTCAAGGCTGAGAAC
Rat MMP-1 sense	GCTTAGCCTTCCTTTGCTGTTGC
Rat MMP-1 antisense	GACGTCTTCACCCAAGTTGTAGTAG
Rat MMP-3 sense	CGGTGGCTTCAGTACCTTTC
Rat MMP-3 antisense	ACCTCCTCCCAGACCTTCA
Rat MMP-13 sense	TGCATACGAGCATCCATCCC
Rat MMP-13 antisense	CTCAAAGTGAACCGCAGCAC
Rat TIMP-4 sense	AGAAGGTAGTCCCTGCCAGT
Rat TIMP-4 antisense	CTTGGCCTTCTCGAACCCTT
Rat *β*-actin sense	CACCCGCGAGTACAACCTTC
Rat *β*-actin antisense	CCCATACCCACCATCACACC

MMP, matrix metalloproteinase; TIMP, tissue inhibitors of matrix metalloproteinase.

**Table 2:** Sequences of siRNA.

siRNA name	Sequence (5′-to-3′)
AKT siRNA sense	GAAGAUCCUCAAGAAGGAATT
AKT siRNA antisense	UUCCUUCUUGAGGAUCUUCTT
NF-*κB* siRNA sense	GGCGAGAGGAGCACAGAUATT
NF-*κB* siRNA antisense	UAUCUGUGCUCCUCUCGCCTT
Negative control siRNA sense	UUCUCCGAACGUGUCACGUTT
Negative control siRNA antisense	ACGUGACACGUUCGGAGAATT

## Data Availability

The data used to support the findings of this study are available from the corresponding author upon request.
